# Formula PSORI-CM01 eliminates psoriasis by inhibiting the expression of keratinocyte cyclin B2

**DOI:** 10.1186/s12906-016-1234-6

**Published:** 2016-07-29

**Authors:** Jian-an Wei, Ling Han, Chuan-jian Lu, Rui-zhi Zhao, Jing Sun, Yue Lu, Han-jie Lin

**Affiliations:** 1 Molecular Biology Laboratory, the Second Affiliated Hospital of Guangzhou University of Chinese Medicine, Guangzhou, Guangdong 510120 China; 2 Department of Dermatology & Guangdong Provincial Key Laboratory of Clinical Research on Traditional Chinese Medicine Syndrome, the Second Affiliated Hospital of Guangzhou University of Chinese Medicine, Guangzhou, Guangdong 510120 China; 3 Pharmaceutics laboratory, the Second Affiliated Hospital of Guangzhou University of Chinese Medicine, Guangzhou, Guangdong 510120 China

**Keywords:** Psoriasis, Keratinocyte, Cyclin, Chinese medicine formula, PSORI-CM01

## Abstract

**Background:**

Psoriasis is a chronically recurrent inflammatory skin disease, modern medicine could achieve good therapeutic effect, but these treatments led to recurrence of the psoriasis, more severe symptoms due to damaging skin barrier. Traditional Chinese medicine is a useful alternative therapies. The purpose of this study was to explore the mechanism of PSORI-CM01, a Chinese medicine formula for psoriasis therapy, in eliminating psoriasis by studying its effects on inhibiting epidermal hyperplasia.

**Methods:**

Imiquimod induced psoriasis-form mice model was used to determine the efficacy of PSORICM-01 by assessing the improvement of hyperplasia in epidermal and dermal skin, cyclin B2 expression in skin was detected by immunochemistry. Human keratinocyte cell line HaCaT stimulated by LPS or not was used to research molecular mechanisms of PSORIMCM-01 as in vitro model. The inhibition of proliferation of HaCaT was determined by MTT assay, BrdU assay and real-time cell analysis (RTCA). Cell cycle distribution was detected by flow cytometry. Real-Time PCR and western blot analysis was performed to quantify the mRNA and protein expression levels, respectively. The ability of PSORICM-01 to inhibit proliferation of cyclin B2 overexpressed HaCaT cell were also investigated.

**Results:**

PSORI-CM01 significantly inhibited epidermal hyperplasia in IMQ mice lesion skin, and reduced expression of epidermis cyclin B2. Serum containing PSORI-CM01 dramatically inhibited keratinocyte HaCaT cell proliferation, no matter stimulated by LPS or not. FACS analysis showed ability of PSORICM-01 to arrest cell cycle in the G2/M phase. Additionally, PSORI-CM01 significant downregulated mRNA and protein expression of cyclin B2, and over-expression of cyclin B2 antagonized the anti-proliferative effect of PSORI-CM01 on HaCaT cells.

**Conclusions:**

PSORI-CM01 inhibits epidermal hyperplasia in imiquimod-induced mouse psoriasis-form model and reduces keratinocyte proliferation in vitro. Our results indicate that PSORI-CM01 may possess therapeutic potential for psoriasis by inhibiting keratinocyte proliferation through downregulation of cyclin B2.

## Background

Psoriasis is a chronically recurrent inflammatory skin disease that is characterized by keratinocyte hyperplasia, inflammatory cell infiltration into the dermis and neovascularization. The symptoms commonly include a reddish scaly rash with itching and flaking skin. In general, psoriasis is induced by multiple factors, such as autoimmunity, environment and infection. This disease is estimated to affect 2–3 % of the general population worldwide [1]. Currently, topical agents such as Calcipotriol, combination of Betamethasone, and systemic medication treatment, et al., were used to treat psoriasis, and could achieve good therapeutic effect. But, all these treatments would damage skin barrier, and led to recurrence of the psoriasis, some even with more severe symptoms. Traditional Chinese medicine is a useful alternative therapies.

PSORI-CM01, a Chinese medicine formula for psoriasis therapy, composed of *Curcuma zedoaria, Sarcandra glabra, DarkPlumFruit, Rhizoma Smilacis Glabrae, Lithospermum erythrorhizon, Paeonia lactiflora, Glycyrrhiza uralensis*, is derived from a prescription invented by Xuan Guowei, a nationally renowned dermatologist in the Guangdong Provincial Hospital of Chinese medicine. It has been used to treat psoriasis for more than 20 years without significant side effects. And a trial on effect of PSORI-CM01 in patients with psoriasis was carried out, and the results showed that reduction rate of PASI50 (psoriasis area and severity index) of psoriasis patients after PSORI-CM01 treatment is 61 % (25/41) [2]. Furthermore, proliferation and activation of keratinocytes affects the occurrence and development of psoriasis. So in the present study, we investigated the mechanism of PSORI-CM01 in eliminating psoriasis by inhibiting skin hyperplasia and proliferation of keratinocytes.

## Methods

### Reagents

Tissue Freezing Medium was obtained from SAKURA (Alphen aan den Rijn, NL), Lipopolysaccharide (LPS), methyl thiazolyl tetrazolium (MTT) and dimethyl sulfoxide (DMSO) were obtained from SIGMA-ALDRICH (St. Louis, MO, USA). α-MEM medium and foetal bovine serum were obtained from GIBCO (GRAND ISLAND, NY, USA). The Elisa kit and BrdU used to measure cell proliferation were from Roche Diagnostics, (Mannheim, Germany), the RT reagent kit and SYBR ® Premix Ex Taq ™ II were from TAKARA (DaLian, Liaoning, China), the RIPA buffer and GAPDH rabbit mAb were from Cell Signaling Technology (Beverly, MA, USA), the BCA Protein Assay Kit was from Thermo Scientific (Wilmington, DE, USA), the Cyclin B2 (CCNB2) rabbit polyclonal IgG was from Santa Cruz (Dallas, TX, USA), the Goat anti-rabbit IgG-HRP was from CALBIOCHEM (Merck KGaA, Darmstadt, Germany), the Cellular DNA content detection kit was from Keygen biotech (NanJing, Jiang Su, China), and pEZ-CCNB2 (Expression Clone of Homo sapiens cyclin B2) and pEZ-CTRL (Negative control of Expression Clone) were Fulengen (Guangzhou, GuangDong, China). Lipofectamine ® LTX & PLUS ™ Reagent was from Life Technologies (Grand Island, NY, USA), and PureYield ™ Plasmid Midiprep System was from Promega (Madison, WI, USA). The imiquimod cream was from Sichuan MingXin Pharmaceutical Company, LTD (Chengdu, Sichuan, China), the Compound Glycyrrhizin Tablets (CGT) were from Akiyama Tablet (Shinagawa-ku, Tokyo, Japan), and the Acitretin Capsules were from Chongqing Win Bond Pharmaceutical Company, LTD (Chongqing, China).

### Animals and cellls

All animals, BALB/c mice (20–22 g), were fed on a standard diet, had free access to water and were housed under standard laboratory conditions.

HaCaT cells, an immortalized epidermal keratinocyte cell line, were cultured in MEMα medium containing 10 % FBS in a 37 °C tissue culture incubator with 5 % CO_2_ (Thermo Scientific, 310, DE, USA).

### PSORI-CM01 tablet and its quality

PSORI-CM01 is mainly composed of *Curcuma zedoaria, Sarcandra glabra, DarkPlumFruit, Rhizoma Smilacis Glabrae, Lithospermum erythrorhizon, Paeonia lactiflora, Glycyrrhiza uralensis,* and their ratio is 3:5:5:5:2:3:2. PSORI-CM01 tablets were derived from PSORI-CM01 extract in the Guangdong Provincial Hospital of Chinese medicine.

The quality standards of PSORI-CM01 extract are as follows. Briefly, chromatography analysis was performed using an Agilent Technologies 1100 series system (Berryville, Virginia, United States) equipped with a UV detector and a Kromasil 100–5 C18 column. The mobile phase consisted of 100 % methyl alcohol and 0.1 % acetic acid. The gradient program was as follows: 0–10 min, (10:90–30:70); 10–40 min, (30:70–40:60). The flow rate was 1.0 ml/min. The eluted compounds were identified by standards, and were detected by characteristic peak area. Isofraxidin, liquiritin and astilbin were detected at 320 nm, and paeoniflorin was detected at 230 nm at room temperature. The injection volume was 10 μL. The contents of the tablets were as follows: 1, paeoniflorin about 0.281 mg/g dried medicinal herbs; 2, isofraxidin about 0.133 mg/g dried medicinal herbs; 3, liquiritin about 0.445 mg/g dried medicinal herbs; 4, astilbin about 1.202 mg/g dried medicinal herbs.

### Preparation and quality of PSORI-CM01-containingserum

Blank serum and PSORI-CM01-containingserum (here after referred to as PSORI-CM01 serum) were derived from 20 SPF SD rats weighing from 220–250 g that were purchased from Guangdong Medical Laboratory Animal Centre. Rats were given food water *ad libitum* and were housed at room temperature of 20–22 °C. The animals were randomly assigned into 2 groups: the control group, receiving saline, and the treatment group, receiving a PSORI-CM01 decoction. PSORI-CM01 was administered by gavage, and the rats received 38.6 g crude drug/kg body weight, 2 times daily for 5 days. Food was withdrawn from the rats 12 h before the last gavage, and blood was drawn from the celiac artery under 10 % chloral hydrate anaesthesia 1.5 h after the last gavage. Blood samples were left at room temperature for 6 h and then centrifuged at 3500 rpm for 15 min. The supernatant was collected and sterile-filtered using a 0.22 μm filter. Lastly, the blood samples were frozen at-30 °C.

The quality standards of the PSORI-CM01-containing serum are as follows. Briefly, chromatography of PSORI-CM01-containing serum was performed using an Agilent Technologies 1100 series system equipped with a UV detector and a Phenomenex C18 column. The mobile phase consisted of 100 % methyl alcohol (B), 100 % acetonitrile (C) and 0.05 % formic acid (D). The gradient program was as follows: 0–5 min, B-C-D (20:5:75)-B-C-D (30:5:65); 5–10 min, B-C-D (30:5:65)-B-C-D (35:10:55); 10–20 min, B-C-D (35:10:55)-B-C-D (40:10:50). The flow rate was 0.8 ml/min, and the eluted compounds were identified by standards, and were detected by characteristic peak area at 275 nm at room temperature. The injection volume was 10 μL. The contents of the PSORI-CM01-containing serum are as follows: 1, paeoniflorin approximately 31.15 ug/mL; 2, isofraxidin approximately 1.07 ug/mL; 3, liquiritin approximately 1.43 ug/mL; 4, astilbin approximately 5.35 ug/mL; 5, rosmarinic acid approximately 9.27 ug/mL.

### Mouse model of psoriasis induced by imiquimod

Mice were anesthetized with 200 μl 0.6 % sodium pentobarbital, and the hair was shaved in a 1.5 cm × 2 cm spot on the back. Mice in all groups except for WT had 50 mg imiquimod (IMQ) smeared onto the shaved areas everyday during the first 2–7 days and every other day for the following 8–20 days. The body weights of the mice were recorded daily.

### Assessment of epidermal hyperplasia

BALB/c mice were randomly divided into 6 groups with 10 mice per group: Normal mice without psoriasis induced by imiquimod (WT); imiquimod (IMQ)-induced mice with saline treatment (NS); IMQ-induced mice receiving 6.88 mg/day glycyrrhizin by gavage (CGT); IMQ-induced mice receiving12.5 mg/day PSORI-CM01 tablets (T1); IMQ-induced mice receiving25.0 mg/day PSORI-CM01 tablets (T2); IMQ-induced mice receiving 50.0 mg/dayPSORI-CM01 tablets (T3). According to the treatment, mice in the 5 groups were administered with 400 μl vehicle or drug solution by daily oral gavage from 8–20 days.

On day 20, mice were sacrificed by cervical dislocation, and the back skin was collected and blocked in paraffin for hematoxylin-eosin (HE) staining to observe pathological features. Pictures were taken after the staining, and the skin area in the same width was calculated according to the area formula = pixels/(horizontal resolution × vertical resolution) using a Zeiss Axio Scope A1microscope (Carl Zeiss, Oberkochen, Germany) using Axio Vision software.

### Immunohistochemistry of epidermis

After antigen retrieval and inactivation of endogenous peroxidase activity, tissue sections were incubated with Cyclin B2 antibody overnight at 4 °C. After washing, the sections were incubated with biotinylated goat anti-rabbit IgG, followed by incubation with a solution of avidin-conjugated horseradish peroxidase. Peroxidase activity was detected using H_2_O_2_/diaminobenzidine substrate solution. The slides were counterstained with hematoxylin solution for 1 min. After dehydration, the tissue was sealed with a universal mount. Controls were prepared in the same manner as detailed for the experimental group, except for the incubation process with primary antibody.

### MTT assay

Cells in the exponential growth phase were seeded at a density of 4,000 cells per well in 96-well plates with 200 μL medium/well. Blank serum or PSORI-CM01-containing serum was added after stimulation by LPS. 20 μL MTT solution (5 mg/ml) was added to each well at the detection time points and incubated for 4 h. The formazan crystals were dissolved with 100 μL DMSO. After all the purple formazan crystals was completely dissolved, the OD value of each well was detected in a Multilabel reader (PerkinElmer, 2030) at a 570 nm wavelength.

### BrdU assay

Results were obtained from 6 separate experiments. Cells in the exponential growth phase were seeded at a density of 4,000 cells/well into 96-well plates with 100 μL medium/well. BrdU was used at a final concentration of 10 μM. BrdU and PSORI-CM01-containingserum were added simultaneously with lipopolysaccharide (LPS), and cells were incubated for 16 h. Medium was removed, and 200 μl fixative was added per well. 30 min later, the fixative was removed, and the anti-BrdU-working fluid was added at room temperature for approximately90min. After washing with PBS,100 μl of the chromogenic substrate was added to each well for 15 min. 25 μl of the stop solution was added to each well, and the absorbance at 450 nm was measured using a Multilabel reader (PekinElmer, 2030, MA, USA).

### RTCA assay

The xCELLigence RTCA system (ACEA, DP, CA, USA) was used to monitor cell viability by measuring the Cell Index (CI) transformed by the electrical impedance between the cell surface and the bottom electrode plate. The CI value is positively correlated with the number of cells and cell vitality. Briefly, cells in the exponential growth phase were seeded at a density of 4,000 cells per well in E-Plate 16 plates with 200 μL medium/well. Data were collected every 30 min, and the total monitoring time was 100 h.

### Analysis of cell cycle

The cells were digested with 0.25 % Trypsin, centrifuged, then fixed in cold 70 % ethanol overnight at-20 °C. The cells were washed with cold PBS and re-suspended to a density of 1.0 × 10^6^ cells/ml. 100 μL RNase A was added into 100 μl cell suspension, and incubated at 37 °C for 30 min. Then, 400 μL PI staining solution was added and incubated at 4 °C in dark for 30 min. Finally, red fluorescence distribution was detected at the 488 nm wavelength in the flow cytometry (Beckman Coulter/FC500 Boulevard Brea, CA, USA).

### Real-time PCR analysis

Total RNA was extracted with TRIZOL reagent according to the manual. A spectrophotometer (Thermo Scientific/NANOdrop 2000, DE, USA) was used to determine the concentration and purity of total RNA by measuring the 260 nm/280 nm absorbance ratio. cDNA synthesis was performed with a PrimeScript^TM^ RT reagent kit according to the manufacturer’s instructions. The volume of the qPCR reactions was 20 μL (2 μl template, 200 nM primers), and each sample was detected in duplicate. The reaction conditions were determined in accordance with SYBR Premix Ex Taq^TM^ reagent instructions, and the melting curves were analysed for production specificity in a Real Time PCR system (ABI, 7500, NY, USA). Primers are shown in detail in Table 1.

**Table 1:** Primers applied in the experiments

gene	primer (F/R)	bp
CCNB1	TCTGGATAATGGTGAATGGACA; CGATGTGGCATACTTGTTCTTG	157
CCNA2	AGCTGCCTTTCATTTAGCACTCTAC; TTAAGACTTTCCAGGGTATATCCAGTC	93
CCNB2	TTGCAGTCCATAAACCCACA; GAAGCCAAGAGCAGAGCAGT	218
CCNB3	GAACCAGCCCAAATGTGTCT; ACAACTCCTTCCCAGTGGTG	162
TGFb1	AGGGCTACCATGCCAACTTC; CCCGGGTTATGCTGGTTGTA	103
GAPDH	GGTCTCCTCTGACTTCAACA; AGCCAAATTCGTTGTCATAC	116

### Western blot

The cells were collected and lysed in lysis buffer on ice, and the protein concentration was measured using a BCA Protein Assay Kit according to the manufacturer’s instructions. The concentration of protein was adjusted to 2 μg/μL with sterile water. For the western blot, the Cyclin B2 IgG primary antibody were diluted 1:1000, and the goat anti-rabbit IgG-HRP secondary antibody were diluted 1:10,000 before incubation. chemiluminescence was added prior to exposure of the blot to detect the targeted protein.

### Over-expression of Cyclin B2

The cyclin B2 over-expressing plasmid pEZ-CCNB2 was transformed DH5α, then it was cultured in the LB medium with Ampicillin for screening. PureYield ™ Plasmid Midiprep System was used to purify pEZ-CCNB2, then it was transfected into HaCaT cells with Lipofectamine ® LTX & PLUS. Next, cyclin B2 expression level in the HaCaT cells was detected by Real-time PCR and western blot.

### Statistical analysis

Statistical data analysis was performed using GraphPad Prism 5, and the results are expressed as the mean ± SD. Differences between two groups were compared using Student’s t test, and *P <* 0.05 indicated a statistically significant difference. Image data are only used to illustrate the trends.

## Results

### PSORI-CM01 tablets reduce loss of body weight

**Fig. 1:**
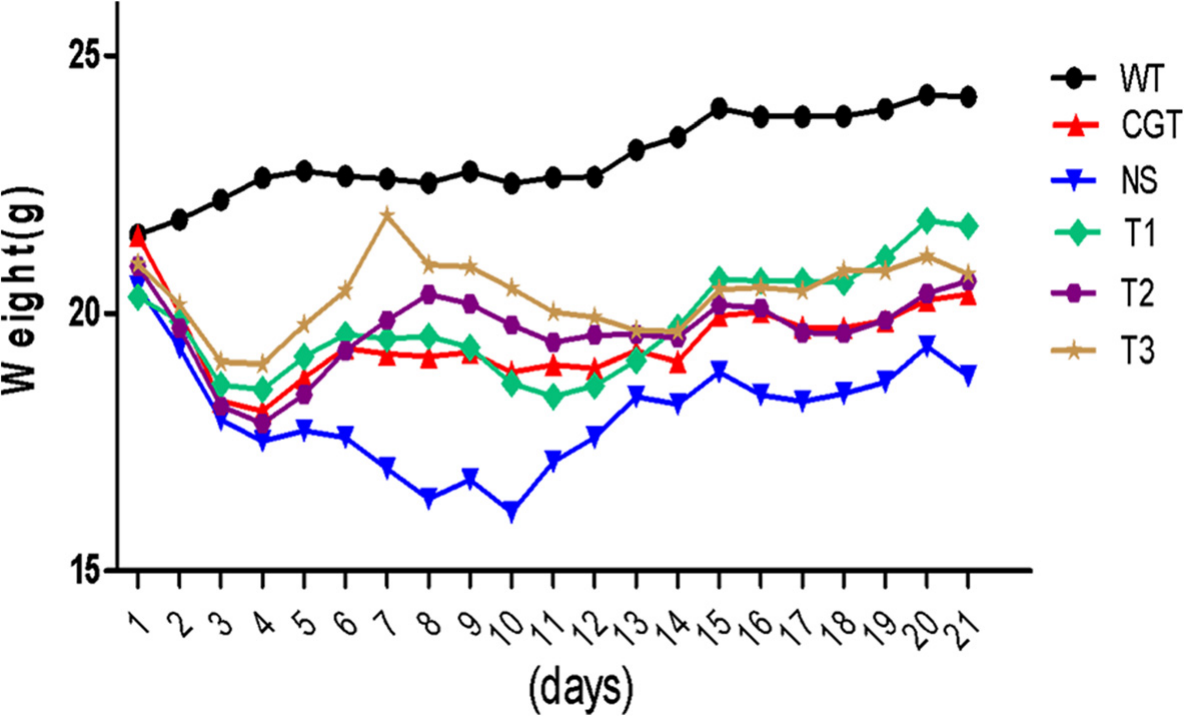
Effect of PSORI-CM01 tablets on body weight in a mouse model of parapsoriasis induced by imiquimod

The body weight of the NS group diminished throughout the experimental period, whereas the body weights of the T1, T2 and T3 treatment groups remained more consistent. The effect of PSORI-CM01 is similar to that of glycyrrhizin in the positive control group (CGT), indicating that the drug therapy improved the overall state of the mice. Detailed results are presented in Fig. 1.

### PSORI-CM01 tablets suppressed epidermal hyperplasia

**Fig. 2:**
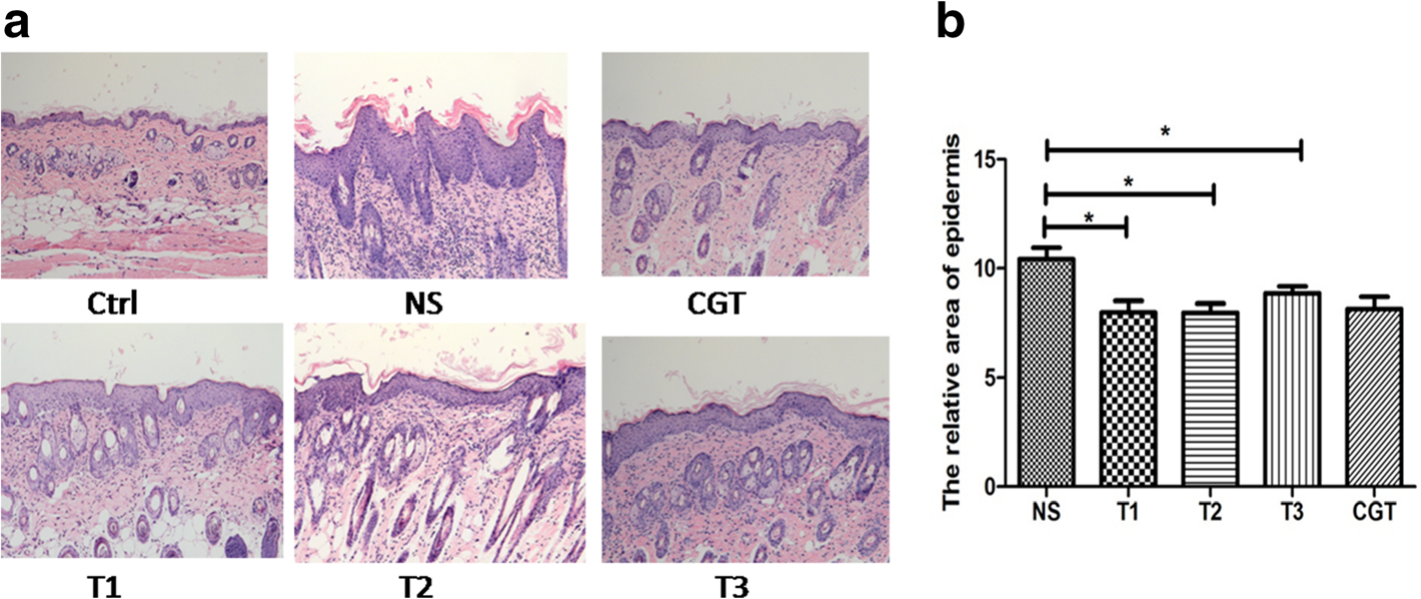
Effect of PSORI-CM01 tablets on epidermal hyperplasia in the mouse model of parapsoriasis induced by imiquimod. **a** Representative HE staining for morphological examination of mouse skin. 400 μl of NS, or 6.88 mg/d CGT, as well as 12.5 mg/d, 25.0 mg/d or50.0 mg/d PSORI-CM01 tablets were administered orally to mice for 12 days after 8 days of imiquimod treatment. Images were captured at 100× (objective lenses 10 ×) under a light microscope using Axio Vision software (Carl Zeiss) at room temperature. **b** Histological sections of the epidermis on day 20 after the first imiquimod treatment, statistical analysis shows the epidermis area relative to the WT group for each group (WT group = 1, *P < 0.05 vs. NS group, *n =* 20)

Typical psoriasis symptoms such as layered scales, erythema, skin wrinkles and thickness were observed in the Imiquimod-induced mouse model of psoriasis. After treatment with PSORI-CM01 tablets, significant changes in skin wrinkles and thickness were observed (*P <* 0.05). Compared with the NS control group, the damaged skin areas of the T1, T2, T3 mice were significantly decreased (*P <* 0.05), and the effect of PSORI-CM01 tablets was similar to that of the positive control group treated with Glycyrrhizin tablets (Fig. 2a, b). These results showed that PSORI-CM01 tablets significantly inhibited epidermal hyperplasia in the Imiquimod-induced mouse model of psoriasis.

### PSORI-CM01 tablets reduced expression of cyclin B2 in the epidermis

**Fig. 3:**
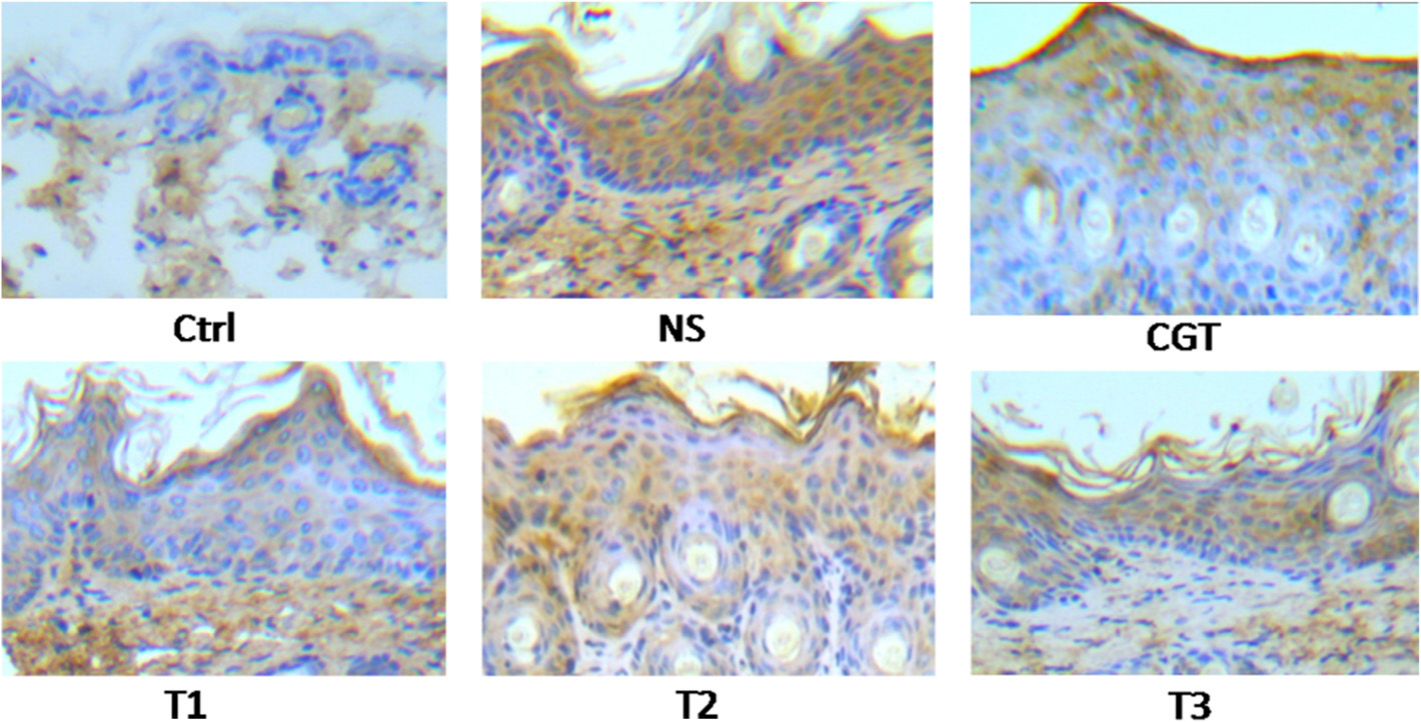
Effect of PSORI-CM01 tablets on cyclin B2 in representative epidermis from each group. magnification ×100. Representative HE staining for morphological examination of mouse skin. 400 μl of NS or 6.88 mg/d CGT were administered orally as a negative and positive control, respectively. 12.5 mg/d, 25.0 mg/d or 50.0 mg/d PSORI-CM01 tablets were administered orally to mice for 12 days after 8 days of imiquimod induction. Images were acquired at 100 × (objective lenses 10 ×) on a light microscope using Axio Vision software (Carl Zeiss) at room temperature

The normal mouse epidermis (WT group) expresses a very low level of cyclin B2. However, the mice in the NS group exhibited an upregulation of cyclin B2 in the epidermis compared with the WT group. Cyclin B2 expression in the epidermis of mice in theT1 group, T2 group andT3 group had a lower staining intensity and frequency compared with that in NS group. The T1 group had the lowest cyclin B2 expression. These results indicate that the PSORI-CM01 tablet inhibited the expression of epidermal cyclin B2 in the Imiquimod-induced mouse model of psoriasis. Representative images of immunohistochemical staining of cyclin B2 are presented in Fig. 3.

### PSORI-CM01 inhibited the growth of HaCaT cells

**Fig. 4:**
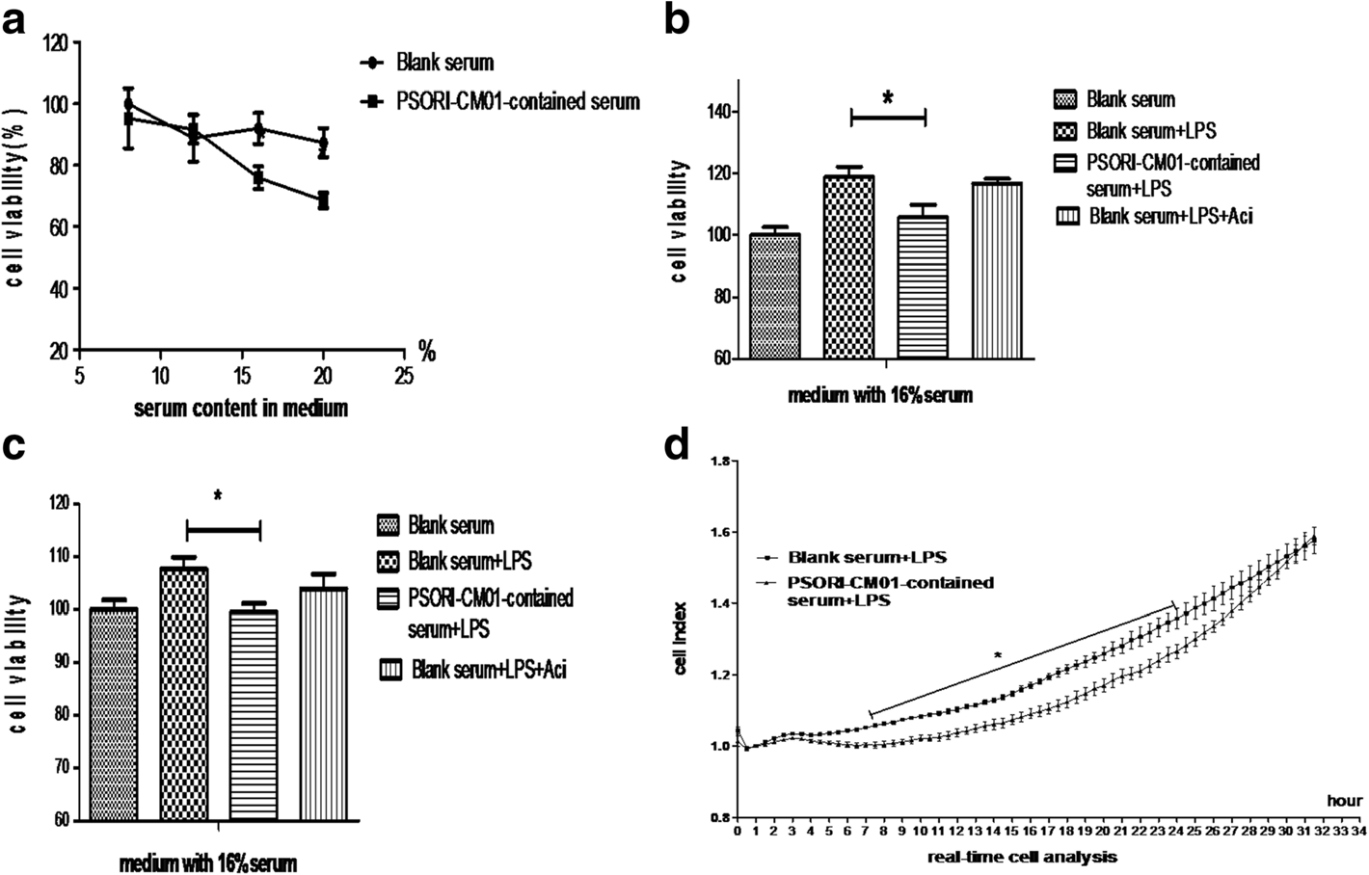
Anti-proliferative effect of PSORI-CM01-containing serum on HaCaT cell proliferation *in vitro* **a** HaCaT cells without LPS stimulation were incubated with various concentrations of blank or PSORI-CM01-containing serum (8 %, 12 %, 16 % and 20 % by volume) for 24 h, then viability was measured using an MTT assay, analysed by measuring the absorbance at 570 nm. **b** HaCaT cells stimulated with 10 μg/ml LPS were incubated with 16 % blank or PSORI-CM01-containing serum for 24 h, then viability was measured using an MTT assay (* *P <* 0.05). **c** HaCaT cells stimulated with10μg/ml LPS were incubated with 16 % blank or PSORI-CM01-containing serum for 15 h, then viability was measured using a BrdU assay, analysed by measuring the absorbance at 450 nm (* *P <* 0.05). **d** HaCaT cells stimulated with10μg/ml LPS were incubated with 16 % blank or PSORI-CM01-containing serum, and then the cell index was measured using the RTCA assay (* *P <* 0.05, each time point during indicated period)

As shown in Fig. 4a, the MTT assay results revealed that the HaCaT cell proliferation was significantly inhibited by PSORI-CM01-containing serum, if the concentration of PSORI-CM01 was above 16 % in the serum (*P <* 0.05). As shown in Fig. 4b and c, the MTT and BrdU assays indicated that the proliferation of LPS-stimulated HaCaT cells was also remarkably inhibited by PSORI-CM01-containing serum (*P <* 0.05). However, the RTCA assay revealed that PSORI-CM01-containing serum inhibited LPS-stimulated HaCaT cell proliferation, and the inhibition lasted more than 25 h, with the most significant inhibitory effect occurring from 8 h point to 25 h point shown in Fig. 4d (*P <* 0.05).

### PSORI-CM01 induced cell cycle G2/M phase arrest

**Fig. 5:**
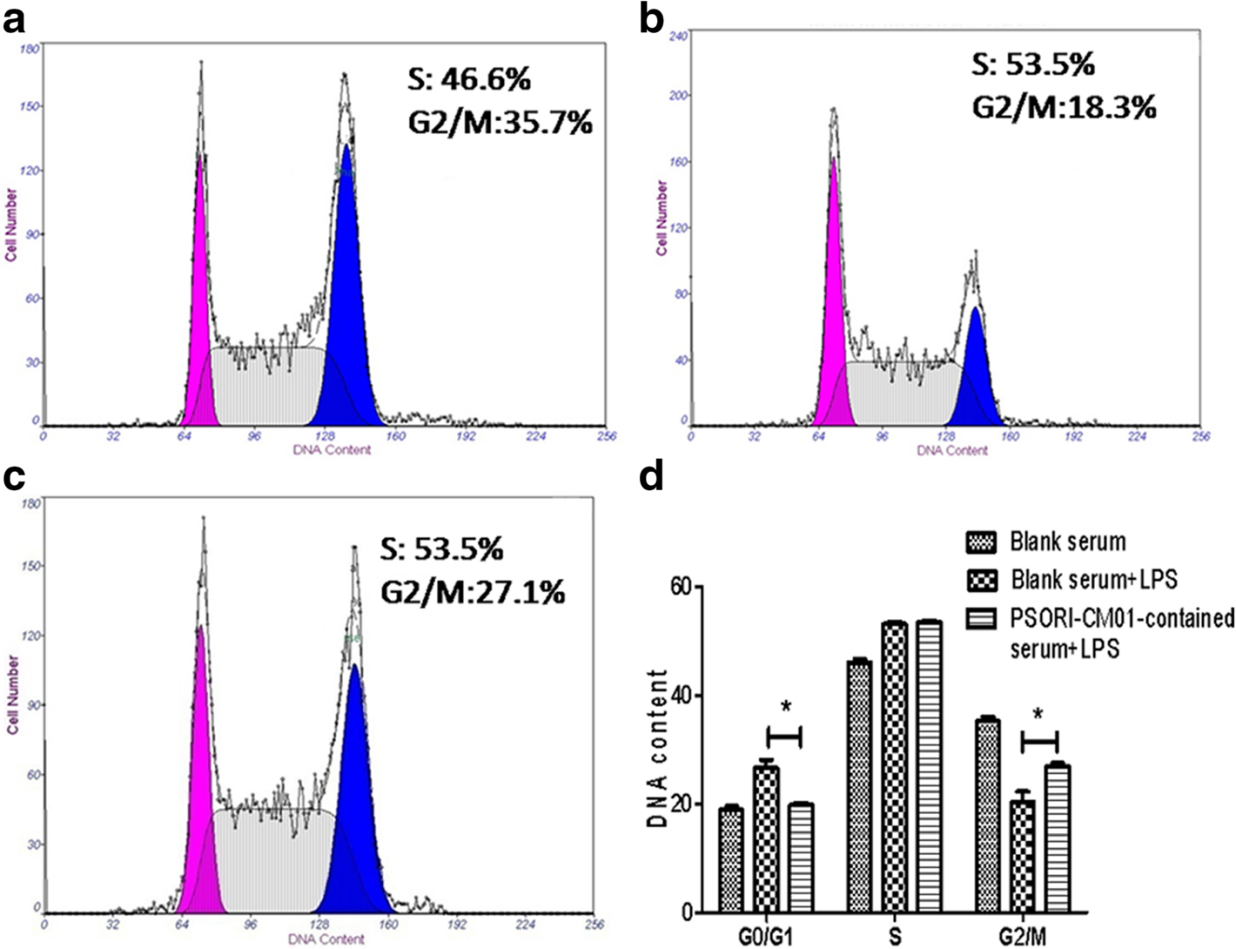
Effects of PSORI-CM01-containing serum on cell cycle. HaCaT cells were stimulated with10μg/ml LPS prior to the addition of 16 % blank or PSORI-CM01-containing serum for 6 h. Cell cycle distribution was measured by flow cytometry. **a**-**c**) Representative image of PI staining by flow cytometry. **d** The cell cycle distribution was quantified in HaCaT cells (with or without LPS stimulation) treated with 16 % blank or PSORICM01-containing serum (*P < 0.05)

We determined the cell cycle distribution of HaCaT cells at different times (6 h, 9 h, 12 h, 15 h, 18 h and 24 h) after treatment with PSORI-CM01-containing serum. As shown in Fig. 5, the results showed a significant arrest of the cell cycle in the G2/M phase both at 6 h and 9 h. Compared with the blank serum, PSORI-CM01-containing serum dramatically decreased the proportion of cells in the G0/G1 phase (*P <* 0.05) and increased the proportion of cells in the G2/M phase (*P <* 0.05), while there was no significant effect on the S phase. These results suggest that inhibition of cell proliferation in LPS-stimulated HaCaT cells may be associated with the induction of cell cycle arrest in the G2/M phase.

### PSORI-CM01 inhibited gene expression and protein level of Cyclin B2

**Fig. 6:**
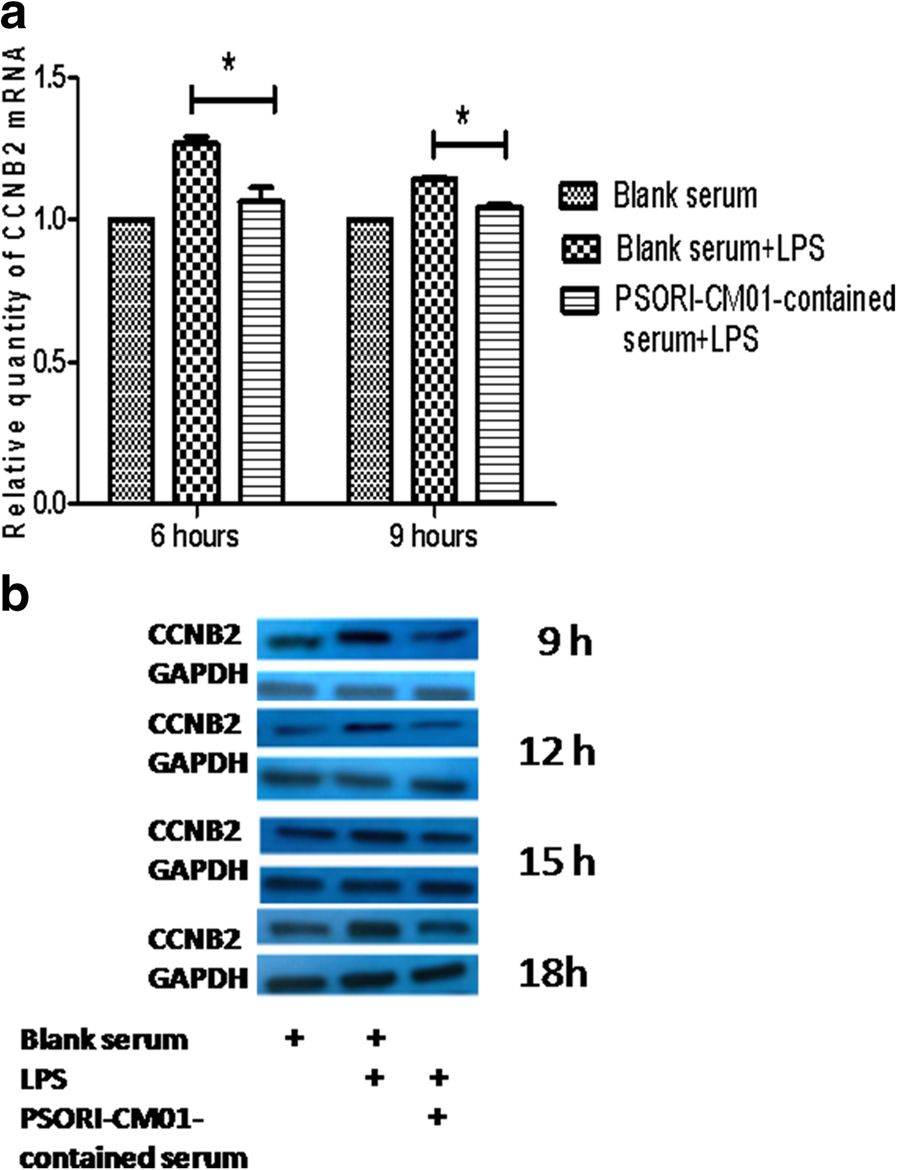
The effects of PSORI-CM01-containing serum on the mRNA expression and protein level of cyclin B2 in LPS- stimulated HaCaT cells. **a** HaCaT cells stimulated with 10 μg/ml LPS were incubated with 16 % blank or PSORI-CM01-containing serum for 6 h or 9 h, then mRNA expression was measured by real-time PCR. **b** LPS-stimulated HaCaT cells were incubated with 16 % blank or PSORI-CM01-containing serum for different times prior to western blot analysis of cyclin B2 expression

Because PSORI-CM01-containing serum arrested HaCaT cells in the G2/M phase, we explored the effects of PSORI-CM01-containing serum on the expression of cell cycle-related gene, including cyclin A2 (CCNA2), cyclin B1 (CCNB1), cyclin B2 (CCNB2), cyclin B3 (CCNB3) and transforming growth factor-beta (TGF-β). Although no remarkable change was observed in the expression of CCNA2, CCNB1, CCNB3 or TGF-β (*P >* 0.05), PSORI-CM01-containing serum significantly inhibited CCNB2 mRNA expression in HaCaT cells stimulated by LPS (Fig. 6a, *P <* 0.05). As shown in Fig. 6b, PSORI-CM01-containing serum significantly inhibited the protein level of CCNB2 in LPS-stimulated HaCaT cells at different times.

### Overexpressed cyclinB2 reversed inhibitory effect of PSORI-CM01 on the proliferation of HaCaT cells

**Fig. 7:**
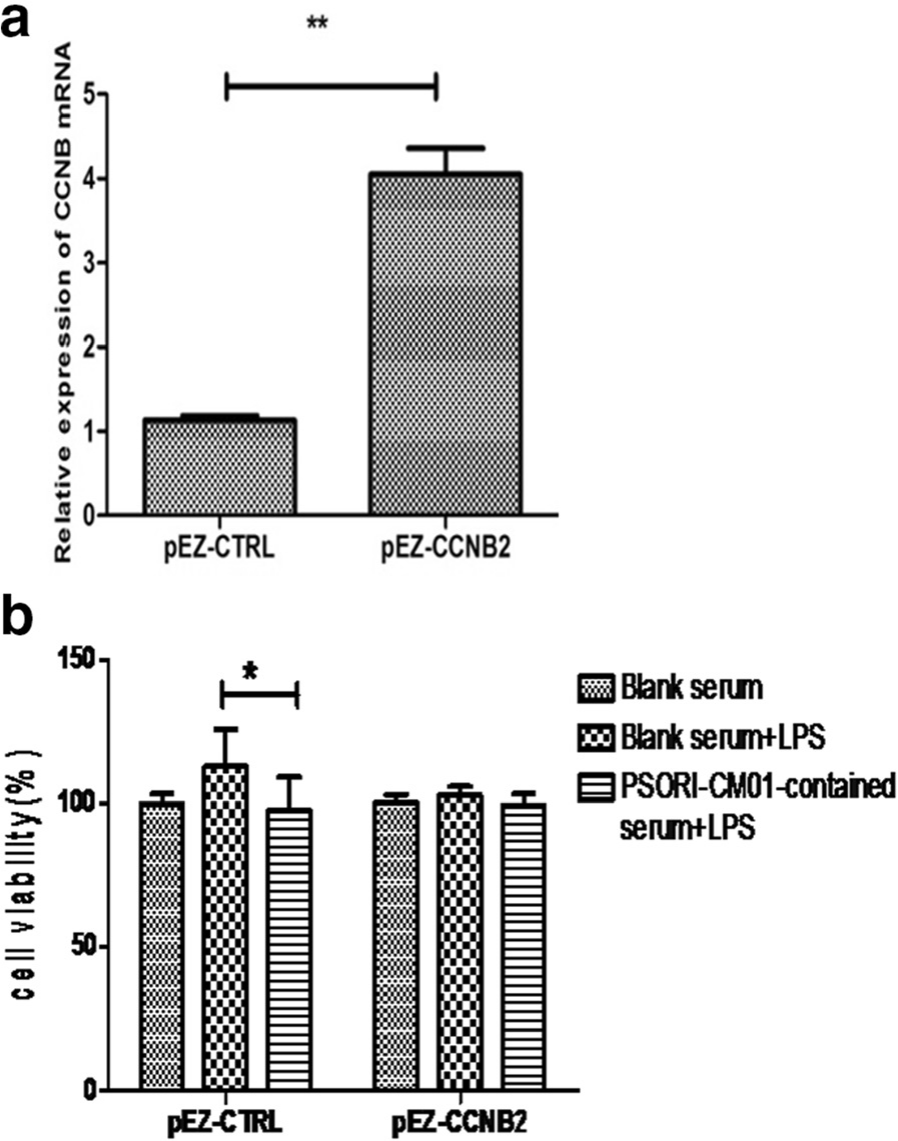
The effects of overexpressed CCNB2 on the proliferation of HaCaT cells treated with PSORI-CM01-containing serum. **a** CCNB2 mRNA was raised significantly in pEZ-CCNB2-transfected HaCaT cell (P<0.05 vs pEZ-CTRL). **b** Treatment with PSORI-CM01-containing serum resulted in significant inhibition of the proliferation of pEZ-CTRL-transfected HaCaT cells stimulated with 10 μg/ml of LPS,however, there was no significant inhibition of proliferation of pEZ-CCNB2-transfected HaCaT cells stimulated with 10 μg/ml of LPS (* *P <* 0.05)

Compared to the pEZ-CTRL transfected control group, the pEZ-CCNB2-transfected HaCaT cells had approximately 4.0-foldhigher mRNA expression (Fig. 7a) and 50 % higher protein expression of cyclin B2. The results of the BrdU assay indicated that PSORI-CM01-containing serum significantly inhibited the proliferation of HaCaT cells transfected with pEZ-CTRL plasmids in LPS-stimulated HaCaT cells (*P* <0.05). However, PSORI-CM01-containing serum did not significantly inhibit the proliferation of LPS-stimulated HaCaT cells transfected withpEZ-CCNB2, as shown in Fig. 7b. These results suggest that overexpression of CCNB2 may reverse the inhibition of PSORI-CM01-containing serum on the proliferation of HaCaT cells.

## Discussion

Psoriasis is a chronic inflammatory skin disease characterized by leukocyte infiltration in the dermis and epidermis, keratinocyte hyper-proliferation, and dilatation and growth of blood vessels. Despite the introduction of new biological therapies has revolutionized the treatment of psoriasis, however, biologics do not cover the needs of those patients whose psoriasis is not severe enough to warrant their use.

PSORI-CM01 is an effective Chinese medicine formula in patients with psoriasis vulgaris. A clinical trial on the formula was carried out in 22 patients with psoriasis vulgaris. The patients were treated with PSOR1-CM01 formula orally, and the observation lasted 2 months. The psoriasis area and severity index (PASI), dermatology life quality index (DLQI) and adverse events were recorded. Compared with those before treatment, PASI scores were significantly decreased after treatment, and DLQI score decreased by 12 % after treatment. No adverse events occurred during the observation. So PSOR1-CM01 formula is considered to be safe and effective for the treatment of psoriasis vulgaris [3], but its mechanism is still unclear.

In the present study, we sought to determine the antipsoriatic properties of Psoricm01, a Chinese medicine that is used for psoriasis vulgaris in Southern China by oral administration, and preliminary clarified its molecular mechanisms. Topical administration of IMQ, a ligand for Toll-like receptors 7 and 8, was reported as a novel mouse model of psoriasis and was used to research pesticide effects and mechanisms of antipsoriatic drugs [4] In this model, mice administrated with PSORI-CM01 tablets had a little loss of body weight compared with the mice in IMQ model group, and Psori-cm01 was also able to ameliorate the course of the disease, resulting in normalization of skin inflammation and keratinocyte proliferation. Then, HaCaT cell line was used as a in vitro tool to justifies the ability of drug serum which contained Psori-CM01 (PSORI-CM01-containing serum) to prevent keratinocyte proliferations. We observed PSORI-CM01-containing serum could obviously inhibit cell proliferation no matter the HaCaT cells were stimulated by LPS or not, when compared with rat control serum.

To further clarify the mechanism of Psori-CM01 growth inhibition, its ability to arrest cell cycle has been studied. The results of FACS analysis showed ability of Psori-CM01 to arrest cell cycle in the G2/M phase. The arrest of cell cycle progression in the G2/M phage is mainly regulated by the maturation promoting factor (MPF). MPF is composed of cyclin B and cyclin-dependent kinase 1 (CDK1). Cyclin B is a key regulated unit that changes cyclically and reaches a peak in expression during the G2/M phase that abruptly diminishes after M phase. While CDK1 activity is dependent on the concentration of cyclin B [5, 6]. In general, inhibition of proliferation and cycle arrest in the G2/M phase is due to the reduction of cyclin B. There are 3 subtypes in cyclin B family, named cyclinB1 (CCNB1), cyclin B2 (CCNB2), and cyclin B3 (CCNB3). We found LPS could enhance CCNB2 mRNA and protein expression in HaCaT cells and Psori-CM01-contained serum could reduce CCNB2 expression both in mRNA and protein level.

To verify the target of Psori-CM01 on HaCaT cell was CCNB2 or not, we further constructed plasmid pEZ-CCNB2 and pEZ-control, transfected to HaCaT cells, and evaluate cell viability stimulated by LPS or not. We found Psori-CM01-contained-serum could not inhibit the hyper-proliferation of HaCaT cells which transfected with pEZ-CCNB2 and stimulated by LPS, meanwhile under the stimulation of LPS, the drug serum showed obviously anti-proliferation effect on HaCaT cells which transfected with pEZ-CTRL plasmid. Our results indicate that inhibit proliferation effect of Psori-CM01-contained-serum on HaCaT cell maybe related to keratinocyte CCNB2 expression. We also detected CCNB2 expression in IMQ mice skin lesion by IHC. CCNB2 was more expressed in psoriasis-form skin especially in the mice epidermis when compared with WT group, while mice administrated with Psori-CM01 had a lower expression of CCNB2 in epidermis. The results showed that CCNB2 abnormally expression maybe associated with IMQ induced psoriasis-form pathological lesion, and Psori-CM01 could inhibit the expression of CCNB2 both in the epidermis in IMQ model in vivo, and in LPS induced HaCaT cell hyper-proliferation model in vitro.

Cyclins were confirmed to could used as a therapy marker for psoriasis. Narrow-band UV-B (NBUVB), a commonly used means of treatment of psoriasis, could obviously reduction the expression of keratinocyte proliferation markers, Ki-67, cyclin A and cyclin B, to 62 %, 68 % and 81 %, respectively after NBUVB therapy [7]. After 6 weeks of cyclosporine therapy, a clinical improvement of the disease and normalization of the epidermis were observed. Epidermal thickness and Ki-67-, cyclin B- and cyclin A-positive nuclei percentage were significantly higher before therapy than after. Whereas cyclin D1 was negative or expressed in a low percentage of nuclei in psoriasis before therapy [8]. Moreover, 1, 25-dihydroxyvitamin D3 (VD3) are also used for the treatment of psoriasis, and it could induce cell cycle arrest not only at the G0/G1 phase but also at the G2/M phase which same with our results. Further research showed VD3 also decreased the expression of cyclin B, some difference from Psori-CM01 reduce expression of CCNB2, VD3 decreased the expression of CCNB1, which forms MPF complex with Cdc2 [9].

In short, our study confirmed PSORI-CM01 formula had a good anti-psoriatic effect for its inhibit hyper-proliferation of keratinocyte both in IMQ induced psoriasis-form mice model in vivo, and in LPS-induced HaCaT cell model in vitro. The molecular mechanism of Psori-CM01 maybe related to reduce the epidermis CCNB2 mRNA and protein expression. Cytokine storm and negative feedback loops between cytokines and chemokines in psoriasis skin were thought as the main course of psoriasis patients lesion recurrence, further research of Psori-CM01 on effect of anti-inflammation and anti-cytokines secretion in psoriasis skin, is still needed to been carried out.

## Conclusions

PSORI-CM01 inhibits epidermal hyperplasia in an imiquimod-induced mouse model of psoriasis and reduces keratinocyte proliferation *in vitro*. Our results indicate that PSORI-CM01 may possess therapeutic potential for psoriasis by inhibiting keratinocyte proliferation through downregulation of cyclin B2.

## Abbreviations

CCNB, cyclin B; CGT, Compound Glycyrrhizin Tablets; DMSO, dimethyl sulfoxide; EDTA, ethylene diamine tetraacetic acid; FBS, fetal bovine serum; HE, hematoxylin-eosin; IMQ, imiquimod; LPS, lipopolysaccharide; MTT, methyl thiazolyl tetrazolium; OD, optical density; PCR, polymerase chain reaction; RTCA, real-time cell analysis
